# Cell extrinsic alterations in splenic B cell maturation in Flt3-ligand knockout mice

**DOI:** 10.1002/iid3.54

**Published:** 2015-04-15

**Authors:** Joseph J Dolence, Kimberly A Gwin, Mariya B Shapiro, Fan-Chi Hsu, Virginia S Shapiro, Kay L Medina

**Affiliations:** 1 Department of Immunology, Mayo Clinic College of MedicineRochester, MN, 55905; 2 Mayo Graduate School, Mayo Clinic College of MedicineRochester, MN, 55905

**Keywords:** B lymphopoiesis, B cell maturation, BAFF, Flt3 signaling, follicular B cells, marginal zone B cells, proliferation, transitional B cells

## Abstract

B lymphopoiesis in bone marrow (BM) is critical for maintaining a diverse peripheral B cell pool to fight infection and establish lifelong immunity. The generation of immature B cells is reduced in Flt3-ligand (*FL-/-*) mice leading to deficiencies in splenic B cells. Here, we sought to understand the cellular basis of the spleen B cell deficiency in *FL-/-* mice. Significant reductions in transitional (TS) and follicular (FO) B cells were found in *FL-/-* mice, and increased frequencies, but not absolute numbers, of marginal zone (MZ) B cells. BAFF-R expression on splenic B cells and serum levels of B cell activating factor (BAFF) was comparable to wildtype (WT) mice. Mixed BM chimeras revealed that the reductions in TS and FO B cells were cell extrinsic. FL administration into *FL-/-* mice restored the deficiency in TS B cells and normalized the MZ compartment. Ki67 analysis revealed a significant decrease in the proliferative capacity of TS B cells in *FL-/-* mice. A Bcl2 transgene did not rescue TS cells in *FL-/-* mice, uncoupling FL-deficiency to Bcl2-dependent survival pathways. Upregulation of CD1d expression and adoptive transfer experiments suggested MZ skewing in *FL-/-* mice. These findings support an integral role for Flt3 signaling in peripheral B cell maturation.

## Introduction

In adult mice, B-lymphocytes are produced in the bone marrow (BM) as one of the lineage progeny of pluripotent hematopoietic stem cells. Upon successful rearrangement of the immunoglobulin heavy and light chain genes, B cell precursors express surface IgM and then are tested for self-reactivity. Non-self-reactive, immature B cells either initiate B cell maturation in the BM or exit the marrow, enter the blood, and travel to the spleen to complete maturation 1. Three major subsets of B cells can be distinguished in the spleen. Transitional B cells are newly produced and short-lived 2,3. They survive only 2–4 days unless selected into one of the other two major splenic B cell subsets, follicular (FO), or marginal zone (MZ) B cells 2,4. FO B cells comprise the major pool of recirculating B cells, while MZ B cells reside in the marginal sinus and in mice, do not recirculate.

Various cellular pathways for B cell maturation in the periphery have been characterized 2–7. TS B cells are IgM^hi^, express the B-cell precursor antigen CD93/AA4, and varying levels of CD21/35. TS B cells are fractionated into two additional subsets, transitional 1 (T1) and transitional 2 (T2). T1 cells are CD21/35^−^, CD23^−^, and IgD^−/lo^, while T2 cells are CD21/35^int^, CD23^+^, and IgD^+/hi^. T1 cells give rise to T2 cells that are the major precursor for FO and MZ B cells. FO B cells are IgM^+lo^, CD21/35^int^, IgD^+^, CD23^+^, and CD24^+lo^, and MZ B cells are IgM^hi^, CD21/35^hi^, IgD^lo^, CD23^−^, and CD1d^hi^.

B cell maturation is dependent on multiple factors including responsiveness to multivalent antigen, BAFF-R signaling, and BCR signaling in order to select non-self-reactive B cells that comprise the long-lived mature B cell pool. T1 B cells are the least mature splenic B cell subset, are in the G_0_/G_1_ phase of the cell cycle, and are nonresponsive to multivalent antigen and B cell activating factor (BAFF). On the other hand, T2 B cells are in cycle and have acquired the ability to robustly respond to multivalent antigen and BAFF 1,4,8. BCR signaling is critical for the transition from T1 to T2 as mice lacking the cytoplasmic tail of Igα have no detectable T2, MZ, or mature B cells 3. In particular, BCR signaling has been hypothesized to induce a proliferative response at this stage necessary for the development of T2 B cells 3. The specific events that drive peripheral B cell maturation downstream of the BCR are unclear, but have been linked to the splenic microenvironment.

The selection of TS B cells into the FO or MZ B cell pools is a critical, yet incompletely understood step in peripheral B cell maturation. Currently, two models have been proposed to mechanistically understand the FO versus MZ cell fate decision: the BCR signal strength model and the production bottleneck model 9,10. The signal strength model posits that if a T2-follicular precursor receives a strong signal via its BCR, then it is induced to differentiate into a FO B cell in a Btk-dependent manner. Alternatively, if the same precursor reacts weakly to antigen, it survives through signals provided by BAFF and weak BCR signaling and becomes competent to inductive signals that promote the MZ B cell fate. The production bottleneck model posits that when B lymphopoiesis in BM is reduced, there is enhanced MZ B cell production. Although the mechanism driving this model is yet to be understood, expansion of the MZ compartment is hypothesized to be necessary to maintain a diverse repertoire of natural IgM.

Microenvironmental signals play a critical role in peripheral B cell maturation. BAFF is a type II membrane protein and a member of the TNF family expressed in either cell bound or soluble forms by radio-resistant stromal cells, monocytes, macrophages, dendritic cells, and T cells, albeit at low levels 11,12. BAFF production is influenced by cytokines including interferon-α, interferon-γ, granulocyte-colony stimulating factor, CD40-ligand, lipopolysaccharide, and peptidoglycans 11. T1 cells express low levels of the receptor for BAFF, BAFF-R, and are largely nonresponsive to BAFF-R signaling 11. In contrast, T2, FO, and MZ B cells are responsive to and dependent on BAFF-R signaling 11. The critical role of BAFF-R signaling in peripheral B cell survival and homeostasis has been determined through combined analysis of mutant and transgenic mice and exogenous administration experiments in vivo and in vitro (reviewed in 13).

Flt3-ligand (FL) is a soluble and membrane bound cytokine produced by stromal cells, hematopoietic progenitors, and activated T cells 14. FL binding to its cognate receptor, Flt3, is critical to establish normal levels of Flt3^+^ multipotential progenitors in BM, maintain the lymphoid progenitor pool, and regulate the number of B cells generated from lymphoid progenitors 15–18. Mice deficient in FL have reduced splenic and lymph node cellularity, reductions in B cells, NK cells, and DCs, and early thymic progenitors 19,20. Flt3 expression is silenced at an early stage in B cell development in BM (after the Pro-B cell stage) and is not re-expressed on splenic B cells (The Immunological Genome Project, http://www.immgen.org/)^21^. Thus, reductions in splenic B lymphocytes in *FL-/-* mice are likely cell extrinsic.

Herein, we document select deficiencies in T1, T2, and FO B cells in *FL-/-* mice. Serum levels of BAFF and cell surface expression of BAFF-R on splenic B cells in *FL-/-* mice were comparable to WT mice, suggesting BAFF-independent regulation. Radiation chimeras confirmed that the deficiencies in TS and FO B cell subsets were cell extrinsic. FL replacement therapy in *FL-/-* mice rescued the TS and FO B cell deficiencies and normalized frequencies of MZ B cells. We show that FL deficiency impairs the proliferation, but not survival of TS B cells. Finally, we provide two pieces of evidence that suggest that FL deficiency skews TS B cell maturation into the MZ B cell fate. First, *FL-/-* mice display an upregulation of CD1d, a hallmark of MZ B cells, starting in T1 cells. Second, WT T1 cells generated an increased frequency of MZ cells when adoptively transferred into *FL-/-* mice in comparison to WT mice. These new data suggest an integral indirect role for Flt3 signaling in regulation of B cell maturation in the spleen.

## Results

### Mice deficient for Flt3-ligand have reductions in TS and FO B cells in the spleen

Flt3 signaling sets the threshold for B lymphopoiesis in BM 15. Consistent with the reduction in B cell precursors in *FL-/-* mice, numbers of immature B cells that have completed the B lineage differentiation program are reduced (Supporting Information Fig. S1). Immature B cells in BM are identified as IgM^+^CD24^hi^ and recirculating B cells as IgM^+^CD24^lo^ 5,6. Enumeration of IgM^+^CD24^lo^ recirculating B cells in the marrow revealed a statistically significant decrease (Supporting Information Fig. S1). This observation prompted further evaluation of peripheral B cell development in *FL-/-* mice.

**Figure 1:**
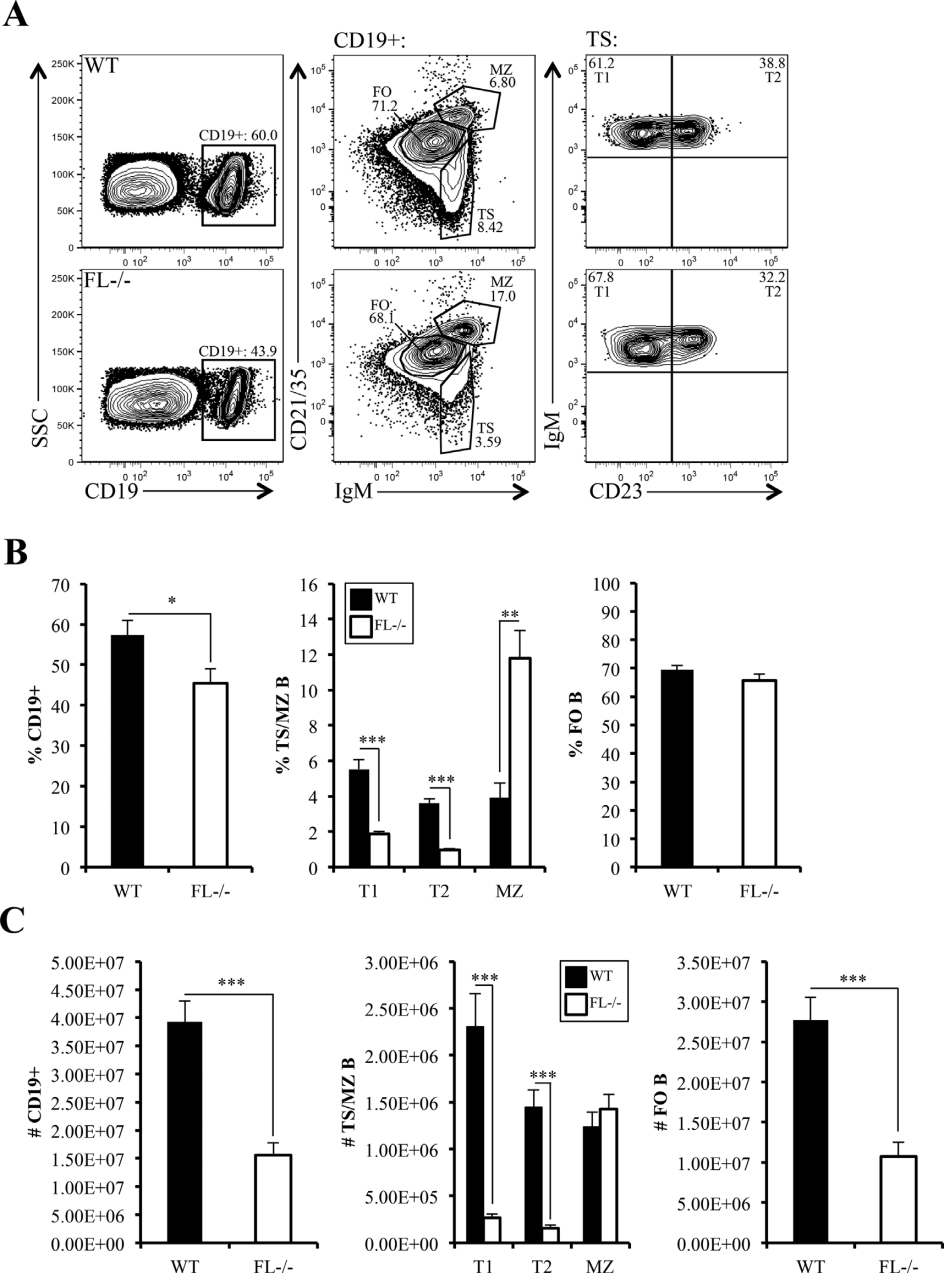
Impaired peripheral B cell maturation in *FL-/-* mice. (A) Flow cytometric analysis of splenic CD19^+^ B cells from a representative wild-type (WT) and *FL-/-* mouse further stained by CD21/35, IgM, and CD23 to examine transitional (TS), marginal zone (MZ), and follicular (FO) B cell subsets. TS B cells are further stained using CD23 to characterize T1 and T2 B cells (*right panels*). (B and C) Bar graphs illustrating the frequency (B) and absolute numbers (C) of total CD19^+^ and T1, T2, MZ, and FO B cell subsets in the spleens of WT and *FL-/-* mice. The bars represent WT (black) or *FL-/-* (white). (B) Frequencies reflect the proportion these cells represent within the CD19^+^ fraction of spleen. (A–C) Data are representative of 15–16 mice/genotype and six independent experiments. Error bars represent mean ± SEM. *, **, and *** represent statistically significant differences measured using the Student's *t*-test at *P *≤ 0.05, *P *≤ 0.005, and *P *< 0.0001, respectively.

Spleen cellularity is reduced in *FL-/-* mice and our results confirmed this finding (1.24 × 10^8^ ± 8.85 × 10^6^ vs. 6.74 × 10^7^ ± 8.42 × 10^6^, WT vs. *FL-/-*, respectively; *P *< 0.0001) 19. Percentages, and to a greater extent, numbers of total CD19^+^ splenic B lymphocytes are reduced in *FL-/-* mice (Fig. 1A–C). TS, FO, and MZ B subsets can be distinguished by differential expression of IgM and CD21/35. Total TS cells include recent emigrants from the BM and are reduced (Fig. 1A, 9.15 ± 0.72% vs. 2.84 ± 0.19% of CD19^+^ cells, WT vs. *FL-/-*, respectively; *P *< 0.0001). T1 cells are discriminated from T2 by differential expression of CD23. Both T1 and T2 subsets are reduced in *FL-/-* mice (Fig. 1A–C). Percentages of FO cells were not affected by FL deficiency, although absolute numbers were significantly reduced, consistent with the reduction in splenic cellularity (Fig. 1A–C). MZ B cells are not reduced by FL-deficiency 22. Indeed, percentages of MZ B cells are significantly increased in *FL-/-* mice (Fig. 1A and B). However, as a consequence of reduced spleen cellularity, absolute numbers of MZ B cells are comparable to WT mice (Fig. 1C). This result is identical for MZ precursors (MZP) (IgM^hi^CD21/CD35^hi^CD23^+^, data not shown) 7. Taken together, these data show selective reductions in TS and FO B splenic subsets in FL-deficient mice.

### Reduced BM B cell output does not explain defective peripheral B cell maturation in *FL-/-* mice

*Hoxa9-/-* and *FL-/-* mice exhibit similar reductions in B lymphopoiesis in BM 23. Therefore, we sought to determine if *Hoxa9-/-* mice had a similar defect in peripheral B cell maturation as *FL-/-* mice. As shown in Table1, TS B cells are reduced in *Hoxa9-/-* mice, but not to the same magnitude as in *FL-/-* mice. TS B cells in *Hoxa9-/-* mice are reduced 40%, while TS B cells in *FL-/-* mice are decreased 70% compared to WT. Furthermore, while slightly elevated, frequencies of MZ B cells in *Hoxa9-/-* mice are not significantly different from WT (Table1). Similar to *FL-/-* mice, *HoxA9-/-* mice displayed no alterations in the frequency of FO B cells (Table1). Thus, reductions in BM B cell output alone cannot explain the peripheral B cell maturation defect in *FL-/-* mice.

**Table:** Frequencies of TS, MZ, and FO B cells in mice deficient for FL or HoxA9

Mice	TS	MZ	FO
Wild-type	9.3 ± 0.9	5.4 ± 0.9	63.1 ± 0.8
*HoxA9-/-*	5.6 ± 0.8*	10.7 ± 2.7	63.3 ± 2.2
*FL-/-*	2.9 ± 0.3*	16.3 ± 1.8*	60.3 ± 2.3

Data represents mean ± SEM of peripheral B cell subsets (% of CD19^+^).

*n* = 6 mice/genotype.

* Significance at *P* ≤ 0.01 between wild-type and *FL-/-* or *HoxA9-/-*.

### Reductions in TS and FO B cell subsets in *FL-/-* mice are not due to defects in BAFF or BAFF-R

**Figure 2:**
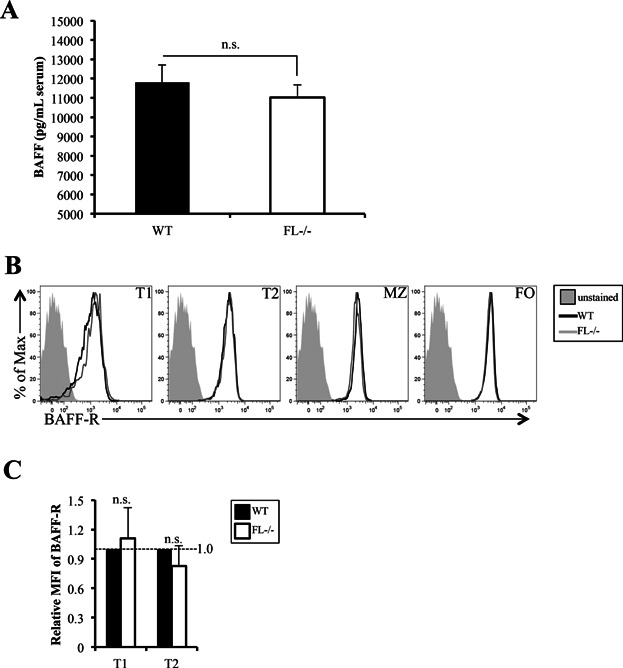
Reductions in TS and FO B cell subsets in *FL-/-* mice are not due to defects in BAFF or BAFF-R. (A) Concentration of BAFF (pg/mL) in the serum of wild-type (WT) and *FL-/-* mice as determined by ELISA. Data is representative of seven mice/genotype. (B) Flow cytometric analysis of BAFF-R in T1, T2, MZ, and FO B cell subsets. The filled histogram represents the unstained control; the black line indicates BAFF-R staining in T1, T2, MZ, or FO subsets in WT mice, and the gray line for *FL-/-* mice. (C) Bar graph depicting the relative mean fluorescence intensity (MFI) of BAFF-R in WT (normalized to 1, further described in Materials and Methods) and *FL-/-* T1 and T2 B cells. The bars represent WT (black) or *FL-/-* (white). (B and C) Data are representative of nine mice/genotype and three independent experiments. (A and C) Error bars represent mean ± SEM. n.s. signifies non-statistical differences measured using the Student's *t*-test (*P *> 0.05) between the means of different genotypes.

Flt3 expression is silenced at the Pro-B cell stage of B cell differentiation in BM and is not re-expressed on splenic B cells (The Immunological Genome Project, http://www.immgen.org) 21. Thus, the reduction in TS and FO B cells is not due to a cell intrinsic defect in peripheral B cell maturation directly regulated by Flt3 signaling. One growth factor important for peripheral B cell homeostasis and selection is BAFF 8,24. Serum levels of BAFF are not altered by FL deficiency (Fig. 2A). BAFF-R expression is upregulated at the T1 to T2 transition and no alteration in surface expression of BAFF-R was observed in T1 or T2 cells in *FL-/-* mice (Fig. 2B and C) 4. Likewise, BAFF-R expression on FO and MZ B cells was not impaired in *FL-/-* mice (Fig. 2B). These data suggest that the reductions in TS and FO B cell subsets in *FL-/-* mice are BAFF-independent.

### Reductions in TS and FO B cell subsets in *FL-/-* mice are cell extrinsic

**Figure 3:**
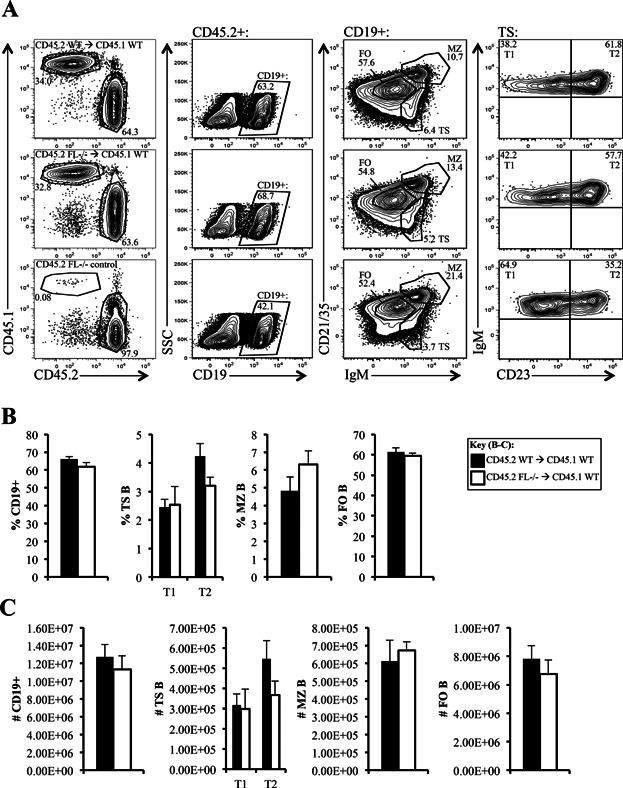
Cell extrinsic defect in peripheral B cell maturation in *FL-/-* mice. (A) Flow cytometric analysis of splenic B cells from a representative host CD45.1 wild-type (WT) mouse that received 2 million CD45.2 WT BM cells (*top panels*) or CD45.2 *FL-/-* BM cells (*middle panels*). CD45.2 *FL-/-* mice served as uninjected controls (*bottom panels*). At 10 weeks, spleens from these mice were harvested and initially analyzed for CD45.1 versus CD45.2 to identify transplanted cells as CD45.2^+^. CD45.2^+^ were further fractionated using CD19, CD21/35, IgM, and CD23 to examine total CD19^+^, TS, MZ, and FO B cell subsets. TS B cells are further stained using CD23 to characterize CD23^−^ T1 and CD23^+^ T2 B cells (*far right panels*). (B and C) Bar graphs illustrating the frequency (B) and numbers (C) of total CD19^+^ and T1, T2, MZ, and FO B cell subsets in the spleens of CD45.1 WT hosts that received CD45.2 WT (black) or CD45.2 *FL-/-* bone marrow (white). Error bars represent mean +/− SEM. (B) Frequencies reflect the proportion these cells represent within the CD19^+^ fraction of spleen. (A–C) Data is representative of five chimeras per group.

Next, we set out to experimentally determine if the reduction in TS and FO B cells in *FL-/-* mice were cell extrinsic. To make this determination, mixed BM radiation chimeras were generated and examined after 10 weeks. CD45.2^+^ WT or *FL-/-* B cells were first gated on CD19^+^, and TS, FO, and MZ B cell subsets were then evaluated within this population (Fig. 3A). As shown in Figure 3A–C, transplantation of *FL-/-* BM cells into WT recipients corrected the deficiencies in TS and FO B cell subsets, and normalized MZ frequencies. Percentages and numbers of total CD19^+^ cells, TS, FO, and MZ B cell subsets in the chimeras were comparable to that generated from CD45.2^+^ WT mice (Fig. 3A–C). We conclude from this experimental data that the defective peripheral B cell maturation observed in the *FL-/-* mice is cell extrinsic.

### Reductions in TS B cell subsets in *FL-/-* mice are corrected by exogenous FL administration

**Figure 4:**
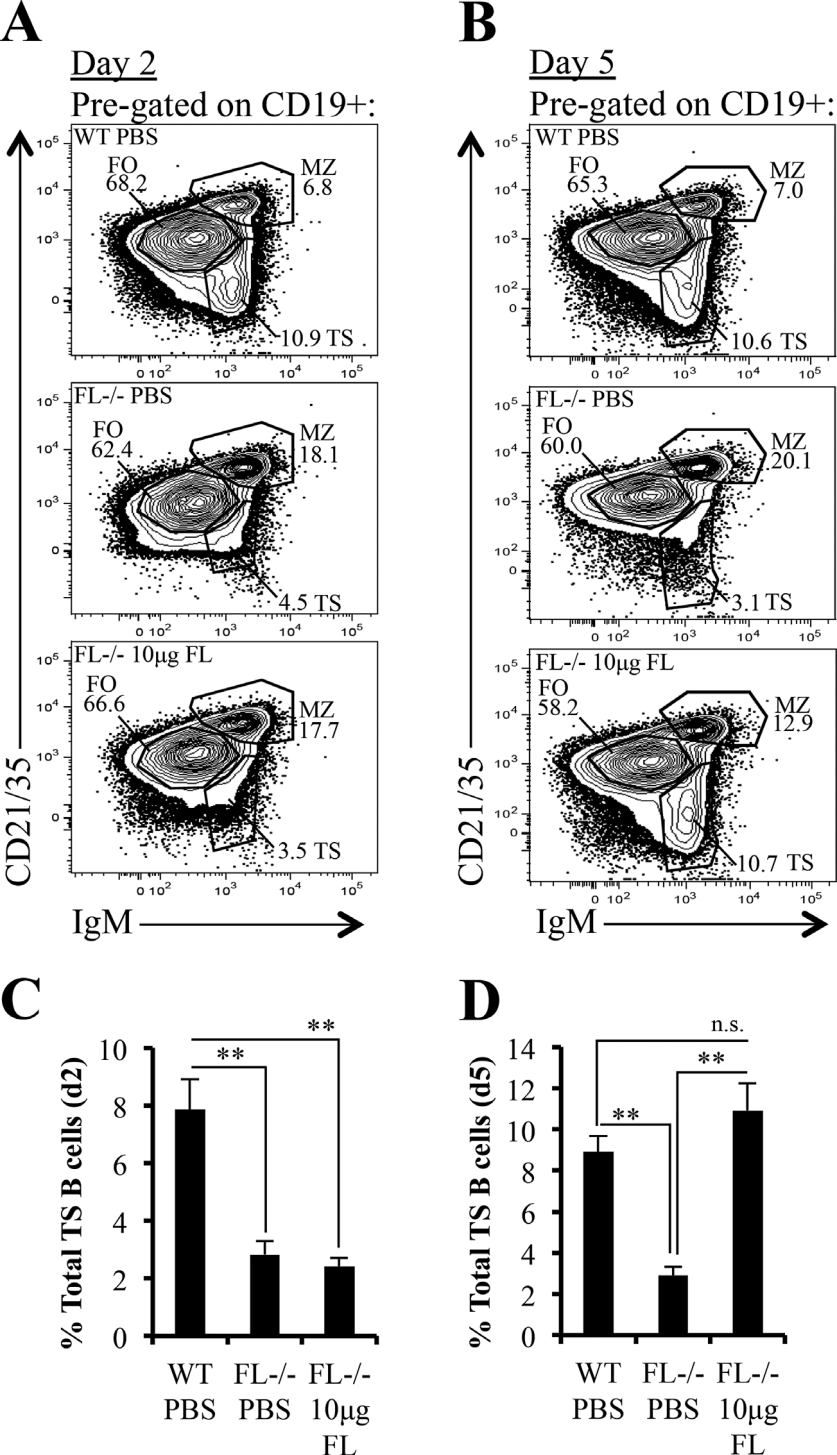
B cell deficiency in *FL-/-* mice is corrected by exogenous FL administration. (A and B) Flow cytometric analysis of spleen taken 2 days (A) or 5 days (B) after either the last injection of PBS (control mice) or FL. Spleen (pre-gated on CD19^+^) is stained with antibodies against CD21/35 and IgM to visualize TS, MZ, and FO B cell subsets in PBS-injected wild-type mice (WT PBS) (*top panels*), PBS-injected *FL-/-* mice (*FL-/-* PBS) (*middle panels*), and FL-injected *FL-/-* mice (*FL-/-* 10 µg FL) (*bottom panels*). (C and D) Bar graphs illustrating the cell frequencies of TS B cells harvested from spleens 2 days (C) or 5 days (D) after either the last injection of PBS (control mice) or FL. (A–D) Data are representative of 4–6 mice/genotype and 2–3 independent experiments. Error bars represent mean ± SEM. ** represent statistically significant differences measured using the Student's *t*-test at *P *≤ 0.005 between the means of different conditions. n.s. signifies non-statistical differences measured using the Student's *t*-test (*P *> 0.05) between the means of different conditions.

We showed reductions in frequencies and numbers of TS B cells in *FL-/-* mice. Next, we evaluated if exogenous administration of FL into *FL-/-* mice was sufficient to restore TS B cells. Ten micrograms of recombinant mouse FL was administered into *FL-/-* mice every other day for 8 days. PBS injected into WT or *FL-/-* mice served as positive and negative controls for FL restoration of TS B cells. On day 2 (Fig. 4A) or day 5 (Fig. 4B) post-FL or PBS injection, splenic B cells were evaluated by flow cytometry. On day 2 following cessation of FL administration, frequencies of TS B cells were unchanged compared to PBS injected *FL-/-* mice (Fig. 4A and C). In contrast, on day 5 following cessation of FL administration, frequencies of TS B cell numbers were substantially increased in *FL-/-* mice (Fig. 4B and D). These data suggest that the TS B cell pool is temporally sensitive to FL-induced alterations to the splenic microenvironment.

### Reductions in percentages of proliferating TS B cells in *FL-/-* mice

**Figure 5:**
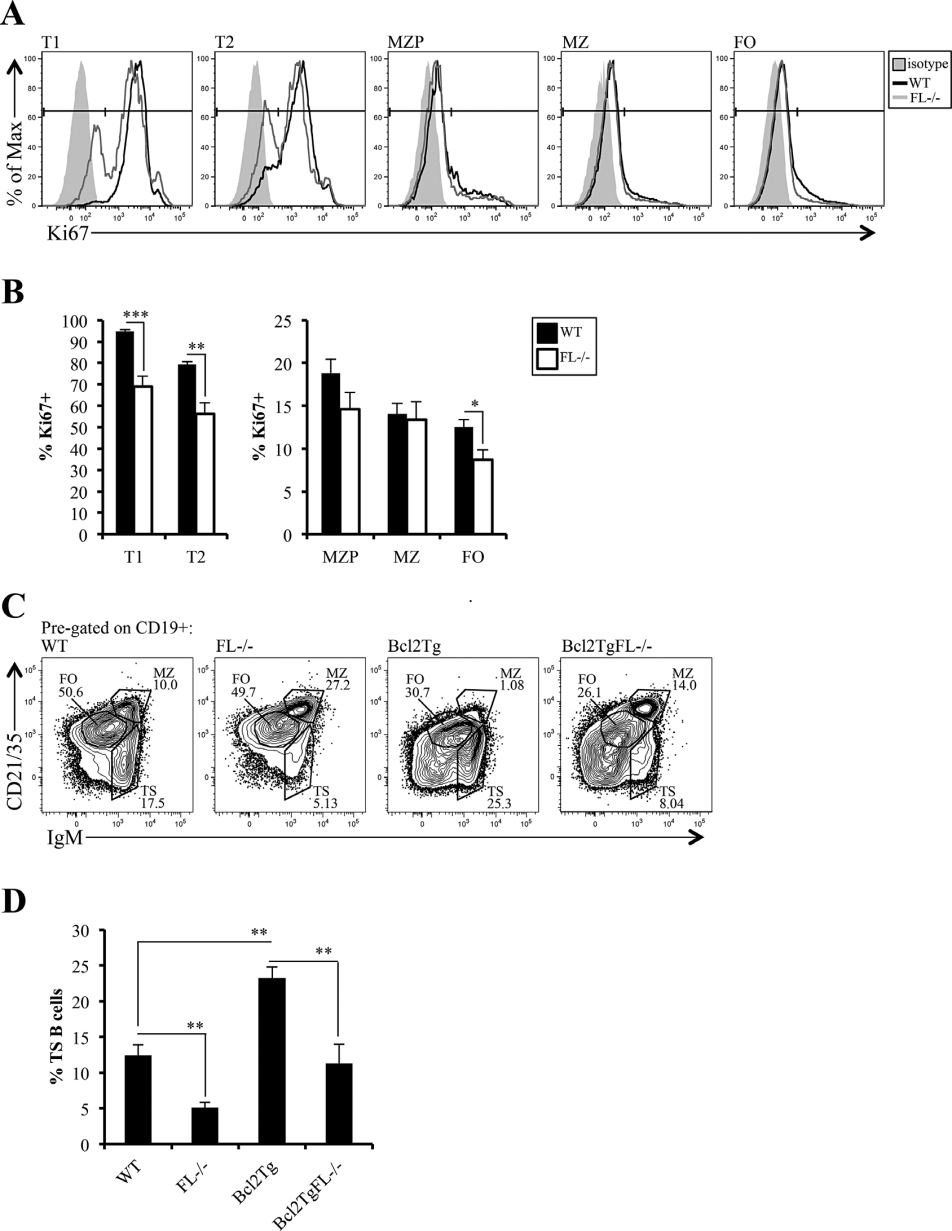
Proliferative capacity, but not survival is impaired in TS B cells in *FL-/-* mice. (A) Intracellular flow cytometric analysis of Ki67 in T1, T2, MZP, MZ, and FO B cell subsets. The filled histogram represents the wild-type (WT) isotype control; the black line indicates Ki67 staining in T1, T2, MZ, MZP, or FO subsets in WT, and the gray line for *FL-/-* mice. *FL-/-* isotype and WT and *FL-/-* unstained controls looked identical to WT isotype control staining (data not shown). T1, T2, MZ, and FO B cell subsets are immunophenotypically defined in Figures 1 and 3. MZP are CD19^+^ CD21/CD35^hi^ IgM^+^ CD23^+^. (B) Bar graphs depicting the frequency of Ki67^+^ cells in WT and *FL-/-* T1, T2, MZP, MZ, and FO B cells. The bars represent WT (black) or *FL-/-* (white). (A and B) Data are representative of 11 mice/genotype and four independent experiments. (C) Flow cytometric analysis (pre-gated on CD19^+^) to examine TS, MZ, and FO B cell subsets in spleens of WT, *FL-/-*, *Eu-Bcl2Tg* (Bcl2Tg), and *Eu-Bcl2Tg FL-/-* (Bcl2TgFL-/-) mice. (D) Bar graph illustrating percentages of TS B cells across the four genotypes displayed in (C). (C and D) Data are representative of 5–8 mice/genotype and four independent experiments. (B and D) Error bars represent mean ± SEM. *, **, and *** represent statistically significant differences measured using the Student's *t*-test at *P *≤ 0.05, *P *≤ 0.005, and *P *< 0.0001, respectively, between the means of different genotypes.

T1 cells are largely comprised of recent emigrants from the BM. Since the generation of immature B cells is reduced in BM of *FL-/-* mice, it was not surprising that the T1 population was reduced. Reductions in non-cycling T1 cells could be presumed to reduce the T2 subset as well. A previous study established that T2 B cells are cycling in vivo 4. We showed above that reduction in TS and FO B cells in *FL-/-* mice was cell extrinsic. To determine if the cell extrinsic defect altered the proliferative capacity of TS and FO B cells in vivo, we compared Ki67 expression in WT and *FL-/-* mice. Ki67 is a nuclear proliferation antigen expressed in G_1_, S, and G_2_ phases of the cell cycle 25. It is not expressed in quiescent or resting cells in G_0_. Importantly, Ki67 expression has been shown to be particularly informative regarding the potential for cellular division, not just the actual cycling state of a given cell 26. Consistent with that interpretation, the vast majority of T1 and T2 cells in WT mice express Ki67 protein, while substantially fewer MZP, MZ, and FO B cells are Ki67^+^ (Fig. 5A and B). Importantly, we document a statistically significant reduction in percentages of T1, T2, and FO B cells in *FL-/-* mice that express Ki67 protein (Fig. 5B). These data indicate that the cell extrinsic defect in *FL-/-* spleen negatively impacts the proliferative capacity of TS, and to a lesser, but statistically significant extent, FO B cells.

### Forced expression of a survival gene does not rescue TS B cells in *FL-/-* mice

Survival signals are critical for peripheral B cell maturation. To determine if the deficiency in TS B cells in *FL-/-* mice is due to impaired survival, we bred *FL-/-* to *Eu-Bcl2Tg* mice 27. Expression of the Bcl2 transgene did not rescue the decrease in splenic cellularity due to FL-deficiency (4.07 × 10^8^ ± 4.67 × 10^7^ vs. 2.53 × 10^8^ ± 6.64 × 10^7^, *Eu-Bcl2Tg* vs. *Eu-Bcl2Tg FL-/-,* respectively; *P *= 0.074). Although it did not reach statistical significance, it was similar (38%) to the decline in splenic cellularity we observed between WT and *FL-/-* (46%). Percentages of TS cells were increased in *Eu-Bcl2Tg* mice compared to WT (Fig. 5C and D). Next, we compared frequencies of TS cells between *Eu-Bcl2Tg* and *Eu-Bcl2Tg FL-/-* mice. We observed a similar reduction in percentages of TS cells between *Eu-Bcl2Tg* and *Eu-Bcl2Tg FL-/-* mice (51%) that we observed between WT and *FL-/-* mice (59%; Fig. 5C and D). Likewise, reductions in absolute numbers of TS cells between *Eu-Bcl2Tg* (2.19 × 10^7^ ± 5.51 × 10^6^) and *Eu-Bcl2Tg FL-/-* (5.21 × 10^6^ ± 3.39 × 10^6^) mice (76%, *P *= 0.036) were comparable to that displayed between WT (1.51 × 10^6^ ± 3.51 × 10^5^) and *FL-/-* (3.20 × 10^5^ ± 4.92 × 10^4^) mice (79%, *P *= 0.0049). These experimental findings suggest that reductions in TS cells in *FL-/-* mice are not due to impaired Bcl2-regulated cell survival pathways.

### Evidence of marginal zone skewing within the T1 and T2 subsets in *FL-/-* mice

**Figure 6:**
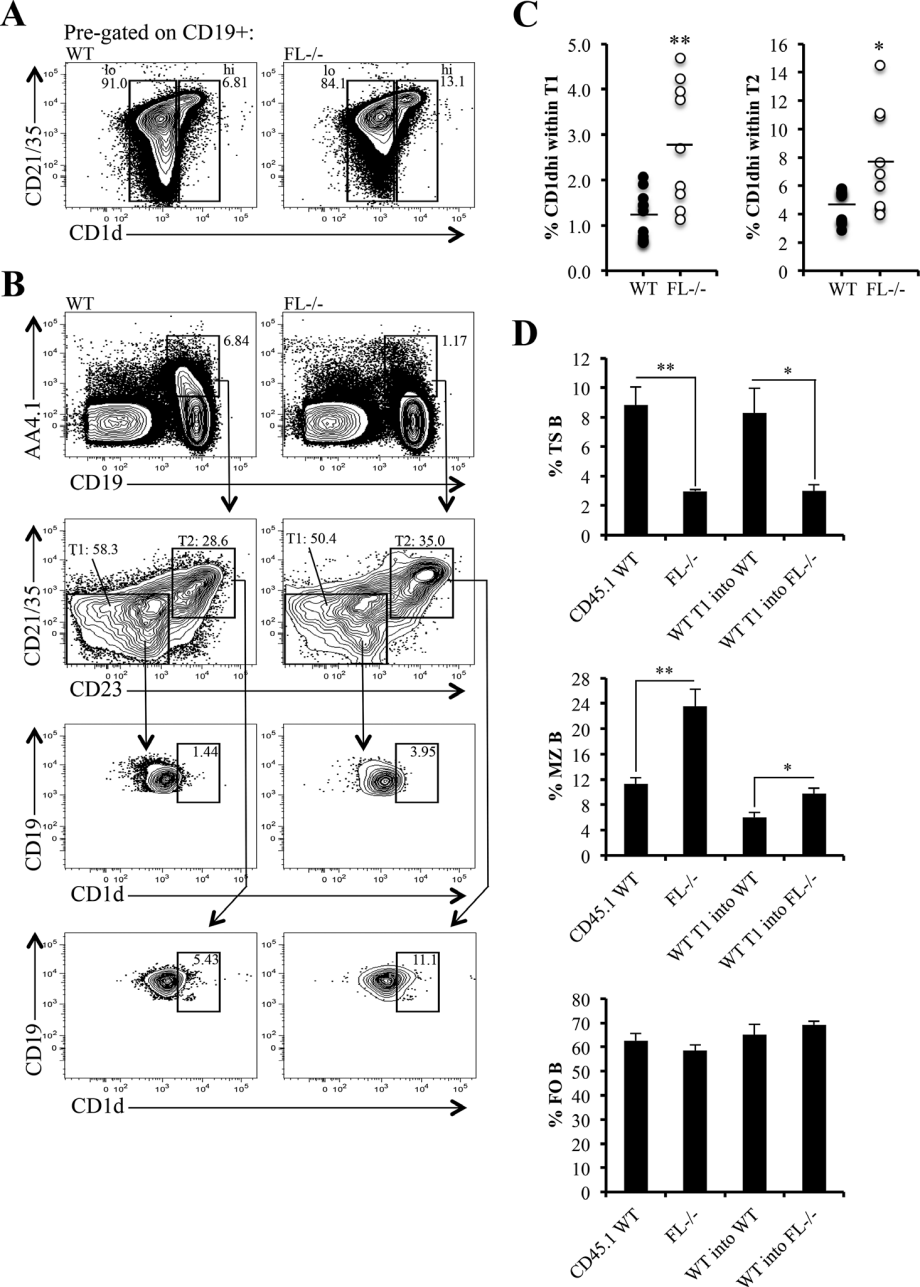
Evidence of marginal zone skewing within T1 and T2 subsets in *FL-/-* mice. (A) Flow cytometric analysis of splenic CD19^+^ B cells (pre-gated) from a representative wild-type (WT) and *FL-/-* mouse further stained by CD21/35 and CD1d to examine CD1d^hi^ and CD1d^lo^ peripheral B cell subsets. (B) Splenic cells were stained using CD19 and AA4.1 to initially resolve CD19^+^ AA4.1^+^ TS B cells. To examine T1 and T2 B cells, CD19^+^ AA4.1^+^ cells were further characterized using CD21/35 and CD23. T1 are defined as CD21/35^-/lo^ CD23^−^ and T2 are CD21/35^lo/int^ CD23^+^ within CD19^+^ AA4.1^+^ TS B cells. T1 and T2 were subsequently analyzed with CD1d to determine the percentage of CD1d^hi^ cells within these TS subsets. CD1d^hi^ gates were set based on expression of CD1d in the MZ as in (A). (A and B) Data are representative of 9–11 mice/genotype and four independent experiments. (C) Scatter plots depict the frequency of CD1d^hi^ cells within T1 and T2 B cells in each WT and *FL-/-* mouse in the analysis (*n* = 11 WT and 9 *FL-/-* mice). (D) Bar graphs illustrating percentages of TS, MZ, and FO B cells generated from WT CD45.1^+^ T1 B cells adoptively transferred into CD45.2 WT (WT T1 into WT) or CD45.2 *FL-/-* (WT T1 into *FL-/-*) mice. The bars labeled CD45.1 WT represent TS, MZ, and FO B cells from uninjected CD45.1 WT mice analyzed to ensure correct CD45.1^+^ and peripheral B cell subset gating. Bars labeled *FL-/-* PBS represent endogenous CD45.2^+^ TS, MZ, and FO B cells from PBS-injected CD45.2 *FL-/-* mice. CD45.1 background staining (controlled by PBS-injected CD45.2 *FL-/-* mice) was very minimal, averaging 0.0003 ± 0.0003% or 12 ± 11 CD45.1^+^ cells (data not shown). Data are representative of 5–9 mice/condition and five independent experiments. (C and D) * and ** represent statistically significant differences measured using the Student's *t*-test at *P *≤ 0.05 and *P *≤ 0.005, respectively.

It has been observed that reduced B cell genesis in BM leads to enhanced MZ B cell production and a corresponding reduction in FO B cells (reviewed in 10). *FL-/-* mice that have reduced production of B cells in the BM faithfully recapitulate this phenomenon (Supporting Information Fig. S1 and Fig. 1). It has been postulated that under conditions of immunodeficiency, humoral mediators involved in homeostatic proliferation and differentiation might increase the MZ cell niche and drive MZ B cell expansion 9. We, therefore, theorized that loss of an appropriate environmental signal due to FL-deficiency might selectively promote MZ B cell development from TS cells. MZP are enriched in the IgM^hi^ CD21/35^hi^ CD23^hi^ fraction of CD19^+^ splenic B cells that express high levels of the glycoprotein CD1d 9,28,29. As shown in Figure 6A, elevated CD1d expression is first detectable in CD19^+^ B cells expressing intermediate levels of CD21/35 then greatly upregulated on MZ B cells that express high levels of CD21/35. *FL-/-* mice displayed an overall increase in frequency of CD1d^hi^ cells, suggesting MZ skewing (Fig. 6A). Next, we sought to determine if skewing of the MZ fate decision occurred in TS cells. TS cells were initially discriminated by expression of AA4.1 and CD19 and further fractionated into T1 and T2 cells by differential expression of CD21/35 and CD23 (Fig. 6B). Within AA4.1^+^ CD19^+^ TS cells, T1 cells were defined as CD21/35^−lo^ CD23^−^ and T2 cells were CD21/35^lo/int^ CD23^+^. In accordance with Figure 1, TS cells were severely reduced in *FL-/-* mice (Fig. 6B). High expression of CD1d has been observed in T2 cells, and CD1d^hi^ T2 cells are presumed MZP 9,29. Therefore, we examined the frequency of CD1d^hi^ cells within T1 and T2 cells to determine whether FL-deficiency skewed TS cells towards the MZ cell fate. As seen in Figure 6B and C, significant increases in the frequency of CD1d^hi^ cells could be detected within the T1 and T2 subsets in *FL-/-* mice. Finally, we conducted adoptive transfer experiments to provide additional evidence that TS cells are skewed towards MZ cell fate in *FL-/-* mice. T1 B cells were sorted from WT CD45.1 mice and adoptively transferred into CD45.2 WT or CD45.2 *FL-/-* mice. Four days later, mice were analyzed to examine the frequency of TS, MZ, and FO cells generated from the transferred WT CD45.1^+^ T1 cells. TS cells survive 2–4 days unless they are selected to become FO or MZ cells 2,4. Therefore, this time point allowed us to determine to what degree the transferred WT CD45.1 T1 cells were maintained as TS cells or became FO or MZ cells. Splenic cells were initially gated using CD45.1 and CD45.2 to identify CD45.1^+^ donor cells. Around twice as many CD45.1^+^ cells were recovered in *FL-/-* than in WT host mice, although this finding was not statistically significant (0.010 ± 0.003% vs. 0.005 ± 0.001% CD45.1^+^ cells, *FL-/-* vs. WT, respectively; *P *= 0.13, data not shown). CD45.1^+^ background staining in CD45.2 *FL-/-* PBS-injected mice was almost non-existent (12 ± 11 cells; 0.0003 ± 0.0003%, data not shown). CD45.1^+^ cells were further gated for CD19, and CD19^+^ cells were fractionated using IgM and CD21/35 to examine TS, MZ, and FO cells. In agreement with our findings that the peripheral B cell maturation defect in *FL-/-* mice is cell extrinsic (Fig. 3), WT T1 cells transferred into *FL-/-* mice were unable to maintain TS cells in comparison to those transferred into WT mice (Fig. 6D). Interestingly, WT T1 cells generated a statistically significant increase in the percentage of MZ cells in *FL-/-* mice than in their WT counterparts (Fig. 6D, 9.71 ± 0.88% vs. 6.03 ± 0.77%, *FL-/-* vs. WT, respectively; *P *= 0.0072), suggesting that TS cells are skewed toward the MZ cell fate in *FL-/-* mice. We found no difference in the ability of WT T1 cells to generate FO cells in WT or *FL-/-* mice (Fig. 6D). Taken together, these data suggest that FL-deficiency preferentially supports the differentiation of MZ B cells once TS cells enter the spleen.

## Discussion

In this study, we show an integral indirect role for Flt3 signaling in regulation of peripheral B cell maturation in the spleen. B lymphopoiesis in BM provides a pool of immature cells that seed the peripheral lymphoid tissues. FL-deficiency reduces production of immature B cells. Numbers of T1, T2 and FO, but not MZ B cells are significantly reduced in spleens of *FL-/-* mice. Peripheral B cell maturation is contingent on BCR signaling, as well as survival and differentiation cues provided by the splenic microenvironment. One cytokine essential for T2, FO, and MZ B cells is BAFF. Serum levels of BAFF in *FL-/-* mice were comparable to WT mice and we found no alteration in surface expression of BAFF-R on splenic B cell subsets. Mixed radiation chimeras together with FL administration experiments confirmed that the reduction in TS and FO B cells was cell extrinsic. Ki67 analysis revealed that FL deficiency altered the proliferative potential of TS, and to a lesser extent FO B cells. Forced expression of Bcl2 did not rescue TS B cells in *FL-/-* mice, suggesting that impaired survival is not the basis of the TS cell deficiency in *FL-/-* mice. Finally, upregulation of CD1d in TS cells, accompanied by an iøncreased frequency of MZ cells generated from WT T1 cells adoptively transferred into *FL-/-* mice suggest that the FL-deficient splenic microenvironment skews TS cells towards the MZ cell fate.

The reductions in TS and FO, but not MZ B cells in *FL*-/- mice are similar to previous observations made in lymphopenic mice 7,10,30. An elegant study by Srivastava et al. showed that transplantation of AA4^+^ T2 or AA4^+^ T1 cells from WT mice into *RAG2-/-* hosts gave rise to MZ-like B cells while the same cells transferred into WT mice generated FO B cells 7. These results were interpreted that in a lymphopenic environment, factors that promote MZ B cell development are more readily available. *FL-/-* mice have deficiencies in TS and FO B cells and thus are lymphopenic compared to WT mice. Similar results were obtained in analysis of *HoxA9-/-* mice that also have reductions in B cell output from BM and reductions in splenic B cells (Table1) 31. Furthermore, we provide evidence that the FL-deficient splenic microenvironment preferentially supports the differentiation of MZ cells once TS cells enter the spleen. One factor essential for MZ B cell differentiation is Notch2 and its ligand, Delta-Like-1 (DL1) 29,32. Presumably, DL1 activation of Notch2 leads to upregulation of Deltex1 in MZ B cells. Notch2 transcripts are expressed at high levels in T2, FO, and MZ B cells 29. We hypothesize that under conditions of FL-deficiency, the microenvironmental signal that limits DL1 activation of Notch2 is reduced. At present, the nature of this signal is unknown. Regardless, TS cells must be highly sensitive to the MZ developmental signal as mild lymphopenia, as we document in *FL-/-* and *HoxA9-/-* mice, is sufficient to promote the MZ B cell fate.

Mixed radiation chimeras established with *FL-/-* BM cells showed normal distributions of TS, FO, and MZ B cell subsets. Frequencies of NK and DCs were normalized as well (data not shown). Moreover, FL administration experiments provided additional evidence that a FL-sensitive component exists in the splenic microenvironment that is necessary for peripheral B cell maturation. Given their well-known requirement for Flt3 signaling, DCs, the only splenic cell type that expresses Flt3, are of particular interest. DCs play a critical role in humoral immunity through production of soluble and cell-bound factors that directly regulate the survival, proliferation, and differentiation of naïve B cells, antigen-activated B cells, and terminally differentiated plasma cells 33–37. However, there are several lines of evidence that suggest that reductions in DCs due to FL deficiency are not the basis of the alterations in splenic B cell subsets we report. First, pDC deficiency as a consequence of targeted inactivation of the transcription factor E2-2 does not impair numbers of splenic B cells 38. Second, CD11c-DTR-EGFP mice allow inducible selective conditional deletion of splenic cDCs upon injection of diphtheria toxin 39. We obtained these mice and effectively depleted splenic cDCs as previously reported and found no alteration in numbers of splenic B cells or alterations in TS or MZ B cell subsets (Dolence et al., unpublished observations). Together, these experimental findings along with our previous report that FL replacement restored the bone marrow B cell deficiency in *FL-/-* mice strongly suggest that the alterations in TS and MZ B cells are the consequence of reduced output of B cells from the bone marrow 18. We note that conditional deletion of FL in mature B cells would strengthen this conclusion, but at present a *FL^fl/fl^* mouse has not been generated.

Due to the importance of BAFF for peripheral B cell homeostasis and selection, it was necessary to examine whether BAFF or BAFF-R was impacted by FL-deficiency 8,24. No differences in serum BAFF or BAFF-R expression were found in *FL-/-* mice. Although DCs, macrophages, and neutrophils are capable of producing BAFF in vitro, BAFF production by radiation-resistant stromal cells is sufficient for B cell survival and maturation 12,40. The selective deficiencies in TS and FO, but not MZ B cells in *FL-/-* mice are consistent with a BAFF-independent role as MZ B cells have a stringent dependence on BAFF 24. Further support for this interpretation is that overproduction of BAFF favors MZ B cell differentiation from T2 cells 41. Finally, since DCs are the only BAFF-producing cell type impacted by FL-deficiency, the finding that indicates defective peripheral B cell maturation in *FL-/-* mice is BAFF-independent is not surprising. We note that we cannot rule out the possibility that local reductions in BAFF, due to diminished numbers of DCs, might contribute to the deficiencies in TS and FO B cells in *FL-/-* mice. Additional experiments, employing transient BAFF replacement, are required to unambiguously rule out BAFF deficiency as a contributing factor in the TS and FO B cell reductions in *FL-/-* mice.

To our knowledge, there has only been a single report of a mouse model that phenocopies the select B cell deficiencies in *FL-/-* mice. Homeodomain-interacting protein Kinase-1 (HIPK1) is a ubiquitously expressed member of the HIPK family comprised of serine/threonine kinases that act as co-repressors for various homeodomain-containing transcription factors 42. *HIPK1-/-* mice exhibit a preferential loss of TS and FO but intact MZ B cells 43. HIPK1 has been implicated in regulation of apoptosis through a p53-dependent pathway 44. Whether deficiencies in HIPK1, in particular the loss of HIPK1-mediated survival signals, contribute to the reductions in TS and FO B cells in *FL-/-* is unknown. However, analysis of *Eu-Bcl2Tg FL-/-* mice indicates that if reductions in TS B cells are due to defective survival, then these signals are Bcl2-independent.

B cells are known to undergo homeostatic proliferation under conditions of lymphopenia. Microenvironmental cues have been suggested to promote homeostatic proliferation, but the exact nature of the signal is unknown. T1 and T2 displayed less proliferative capacity as measured by intracellular Ki67 expression in *FL-/-* mice. This is an intriguing finding and whether the impaired proliferative capacity influences the differentiation capacity of TS cells will be interesting to investigate. Much remains to be learned concerning the microenvironmental signals that dictate the FO versus MZ B cell fate decision. The *FL-/-* mouse provides a unique model to identify the nature of these signals.

## Materials and Methods

### Mice

Wild-type (C57Bl/6) mice were obtained from The Jackson Laboratory (Bar Harbor, Maine). *FL-/-* mice were obtained from Taconic Farms (Germantown, New York), then bred and maintained in our colony. PCR sequences and conditions for genotyping *FL-/-* mice have been described 15. *HoxA9-/-* mice have been described 31. B6-Ly5.2 congenic mice (CD45.1 WT) obtained from National Cancer Institute (NCI) (Frederick, Maryland) were used as recipients to establish mixed bone marrow radiation chimeras. CD45.1 WT mice (obtained from NCI) were also used as donors for adoptive transfer experiments, as T1 B cells were sorted and transferred into CD45.2^+^ WT or *FL-/-* mice. *Eu-Bcl2Tg* mice were kindly provided by Paul W. Kincade (Oklahoma Medical Research Foundation, Oklahoma City, Oklahoma) 45. *Eu-Bcl2Tg FL-/-* mice have been described 18. All mice in this study have been maintained on a C57Bl/6 genetic background for greater than 10 generations. Age-matched or littermate controls were used for individual experiments, and all mice analyzed in this study ranged from 8–12 weeks of age. All animals were bred and maintained at the Mayo Clinic animal facility and experiments were carried out with the approval of the Mayo Clinic Institutional Animal Care and Use Committee.

### Flow cytometry

Methods for flow cytometry have been described 15,46–48. BM was harvested and stained with the following combination of antibodies: B220 APC, IgM APC-Cy7, and CD24 PE. Spleen was harvested, lyzed with ACK to remove red blood cells, and stained with combination of the following antibodies: CD19 (FITC, APC, and PE-Cy7), IgM (FITC, APC-Cy7), CD21/35 (PE, PerCP-Cy5.5), AA4.1 PE, CD23 (PE-Cy7, bio), BAFF-R APC, and CD1d FITC. Staining with CD45.1 PE-Cy7 and CD45.2 FITC antibodies were used to analyze the degree of chimerism of mixed bone marrow chimeras. Staining with CD45.1 PE and CD45.2 FITC antibodies were used to identify the CD45.1^+^ transferred cells in CD45.2^+^ hosts in adoptive transfer experiments. Incubation with streptavidin-APC or streptavidin-APC-eFluor 780 was used to visualize biotin-labeled CD23. All antibodies were obtained from eBioscience (San Diego, California), BD Biosciences (San Jose, California), or BioLegend (San Diego, California). For intracellular Ki67 staining, cells were initially stained for surface antigens (described above), followed by fixation and permeabilization using the Foxp3 staining buffer set (eBioscience) according to manufacturer's instructions. Ki67 or isotype control staining was performed using the FITC mouse anti-human Ki67 set per kit instructions (BD Biosciences). Flow cytometric analysis was performed on the LSRII or Canto cytometers (BD Biosciences). Data were analyzed with FlowJo software (Tree Star, Ashland, Oregon).

### ELISA

The serum concentration of BAFF was calculated by ELISA using the mouse BAFF Quantikine ELISA kit (R&D Systems, Minneapolis, Minnesota). Serum was diluted 10-fold to fall in range of kit detection. The sensitivity for BAFF was 7.8 pg/mL.

### Mixed bone marrow radiation chimeras

Chimeras were established by retro-orbitally transplanting either 2 million C57Bl/6 WT (CD45.2 WT) or *FL-/-* (CD45.2 *FL-/-*) BM cells into irradiated male B6-Ly5.2 congenic mice (CD45.1 WT). Mice were analyzed at 10 weeks after transplantation.

### Flt3 ligand replacement therapy

Methods for Flt3 ligand replacement therapy have been previously described 18. Briefly, two cohorts of *FL-/-* mice were administered 10 µg Flt3 ligand (in 200 µL PBS) every other day for a total of five injections over 8 days. Control WT and *FL-/-* mice were injected with equivalent volumes of PBS. Two days following the last injection, one cohort of mice were euthanized, spleen harvested, and peripheral B cell subsets were analyzed by flow cytometry. The second cohort of mice was euthanized and spleen harvested 5 days following the last injection for flow cytometric analysis.

### Adoptive transfer of T1 B cells

WT T1 B cells (CD19^+^ AA4.1^+^ CD21/35^−lo^ CD23^−^) were sorted from male B6-Ly5.2 congenic mice (CD45.1 WT) and retro-orbitally adoptively transferred into C57Bl/6 WT (CD45.2 WT) or *FL-/-* (CD45.2 *FL-/-*). An average of 5.0-6.0 × 10^5^ WT T1 B cells were adoptively transferred in 200 µL PBS into each of the CD45.2^+^ hosts per experiment. Mice were analyzed 4 days following adoptive transfer. Uninjected CD45.1 WT mice were analyzed along side mice that received T1 B cells to ensure correct CD45.1^+^ and peripheral B cell subset gating. CD45.2 *FL-/-* mice injected retro-orbitally with 200 µL PBS served to control for CD45.1 background staining.

### Cell frequency, absolute number, and mean fluorescence intensity calculations

Cell subset frequencies were calculated by multiplying percentages of sequential gated populations. Absolute numbers were calculated by multiplying mononuclear cell counts obtained after BM or splenic harvest by cell subset frequencies. The frequencies and number calculations reflect mononuclear and doublet exclusion gates. A mean MFI was calculated for WT and *FL-/-* BAFF-R by averaging the MFI of BAFF-R from T1 and T2 B cell subsets from nine WT and nine *FL-/-* mice. The relative MFI of WT BAFF-R was normalized to 1 by dividing the WT mean MFI against itself, and relative MFI of *FL-/-* BAFF-R was calculated by dividing the mean *FL-/-* MFI by the mean WT MFI.

### Statistical analysis

Statistics were performed using the unpaired Student's *t-*test. *P* values ≤ 0.05 were significant. All numerical data are presented as mean ± SEM.

## Supporting Information

**Figure S1**: Immature and recirculating B cells are severely reduced in the BM of *FL-/-* mice.
