# Supplementary figures and images for: Cell extrinsic alterations in splenic B cell maturation in Flt3-ligand knockout mice
Source: Immun Inflamm Dis. 2015 Apr 15;3(2):103–17. doi: 10.1002/iid3.54 (PMC4444153; doi:10.1002/iid3.54)

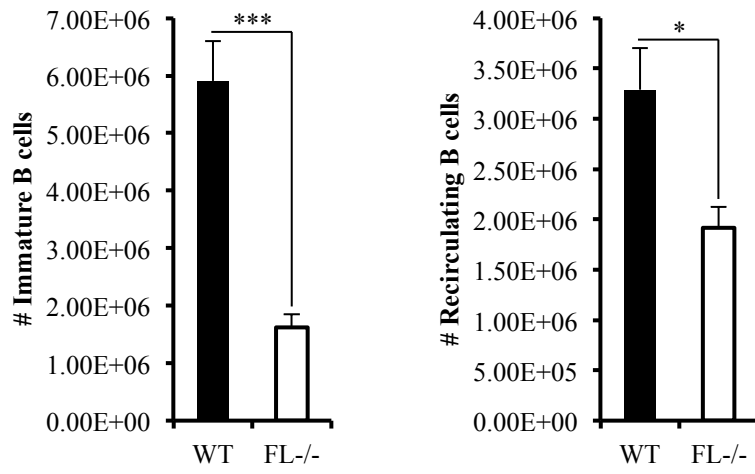

Supplement: Supplementary file 1 [file iid30003-0103-sd1.pdf]
